# Large photocurrent density enhancement assisted by non-absorbing spherical dielectric nanoparticles in a GaAs layer

**DOI:** 10.1038/s41598-020-74186-7

**Published:** 2020-10-13

**Authors:** Bhaskar Singh, Mohammed M. Shabat, Daniel M. Schaadt

**Affiliations:** 1 Institute of Energy Research and Physical Technologies, Clausthal University of Technology, Leibnizstraße 4, 38678 Clausthal-Zellerfeld, Germany; 2 Physics Department, Islamic University of Gaza, P. O. Box 108, Gaza Strip, The Palestinian Authority

**Keywords:** Photovoltaics, Nanoparticles, Nanophotonics and plasmonics

## Abstract

Herein, we report a theoretical investigation of large photocurrent density enhancement in a GaAs absorber layer due to non-absorbing spherical dielectric (SiO_2_) nanoparticles-based antireflection coating. The nanoparticles are embedded in a dielectric matrix (SiN) which improves the antireflection property of SiN ($\lambda /4$ coating) and let to pass more photons into the GaAs layer. The improvement is noticed omnidirectional and the highest is more than 100% at 85° angle of incidence with the nanoparticles’ surface filling density of 70%. Sunrise to sunset calculation of normalized photocurrent density over the course of a year have also shown improvements in the nanoparticles’ case.

## Introduction

Nanoparticles (NPs) are being used extensively in solar cell applications due to its high forward to backward scattering^1–6^. This is caused by suppression of light reflection via high refractive index substrate (famously known as Kerker effect)^7^. Nanostructures such as NPs with size greater than and/or of the order of incident sunlight wavelength show Mie scattering. Additionally, metal NPs also show plasmonic scattering due to the oscillation of free electrons at metal-dielectric interface^1,8^. However, metal NPs face high absorption at the resonance wavelength. On the other hand, dielectric NPs do not show this behavior in a narrow band range^9^. This have captured the attention of scientific community recently and being investigated widely. Dielectric NPs at the front of solar cells have been demonstrated experimentally^10–15^ and theoretically^8,16–19^ with improved device performance. Wan et al.’s TiO_2_ NPs coated solar cell exhibited ca. 30% enhancement in photocurrent^13^. Whereas, Ha et al. demonstrated experimentally more than 30% efficiency gain by using SiO_2_ NPs on a GaAs solar cell^14^. These NPs were closely packed and worked as an antireflection coating (ARC). The wide-angle improvement was also noticed for the entire visible spectrum. Huang et al. measured the normalized current density of with/without sub micrometer silica (SiO_2_) spheres coated amorphous silicon (a-Si) solar cells and noticed the enhancement after 40° angle of incidence (AOI)^20^.

**Figure 1:**
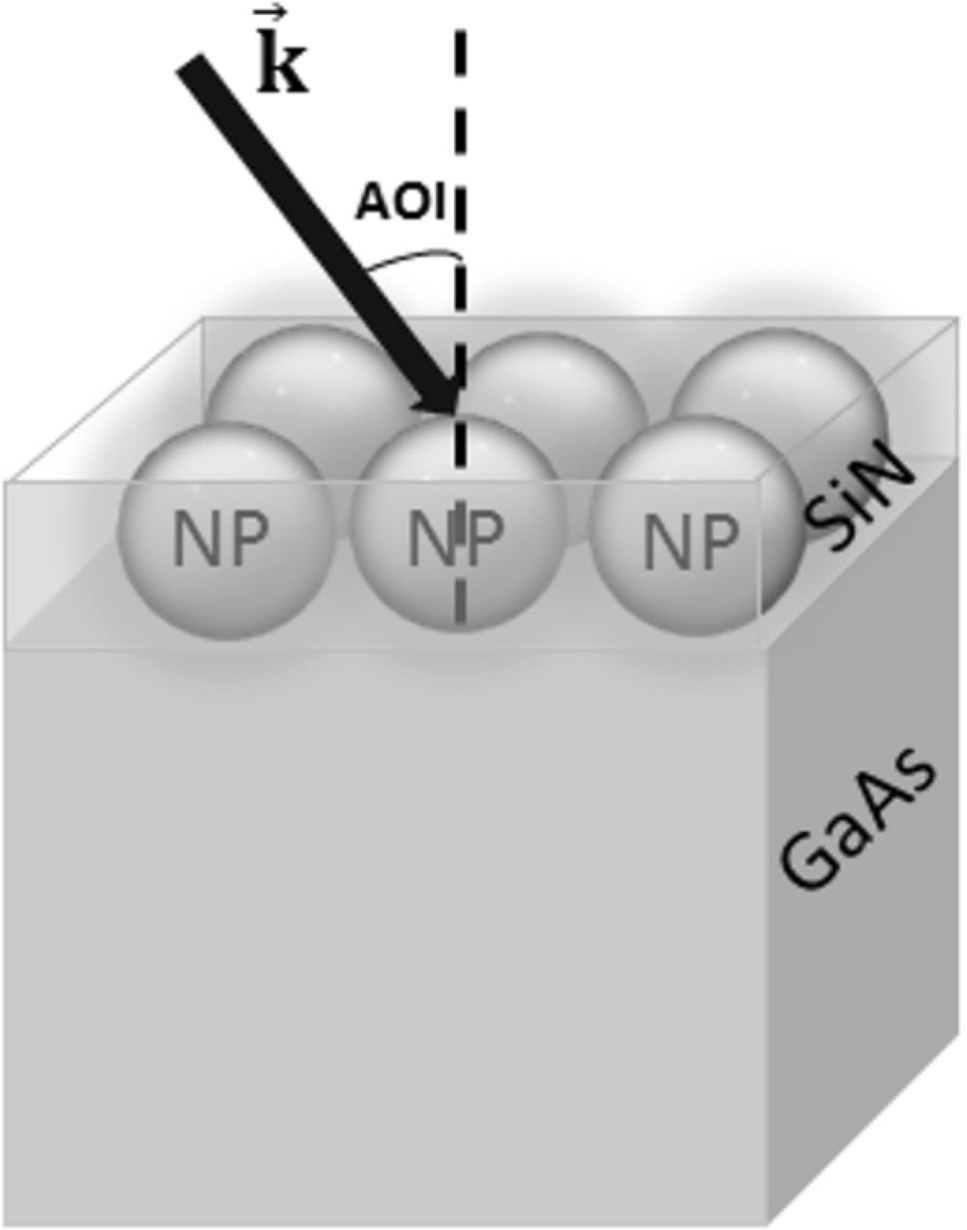
Schematic diagram of the simulated structure.

In this manuscript, we perform an analytical modelling on a structure in which SiO_2_ NPs are embedded in a dielectric ARC (SiN), also called $$\lambda /4$$ coating, on a GaAs layer. The new ARC structure with NPs is called hybrid ARC. We use a dipole model to describe the diffuse reflectance/transmittance behavior of NPs^21^. However, the specular behavior of SiN ARC layer is characterized by Abelès’ famous transfer matrix method (TMM)^22^. The analytical model is presented in detail elsewhere^23^. The hybrid ARC made of two-dimensional (2D) array of the SiO_2_ NPs of equal period in the dielectric SiN matrix bounded with air and a GaAs absorber layer was investigated, as shown in Fig. 1. All the layers and NPs have wavelength dependent index of refraction which is taken from Palik for the calculation^24^. AOI varies from normal incidence (0°) to 85°. NPs’ surface filling density $$\left( f \right)$$ is calculated by $$f = N*\left( {\pi r^{2} } \right)/\left( {l*b} \right)$$, where $$N $$ is the total no. of embedded NPs; $$r$$ is the radius of NPs;$$ l$$ is length, and $$ b$$ is breadth of SiN layer. The dipole approximation is valid for $$f < 0.75$$.

**Figure 2:**
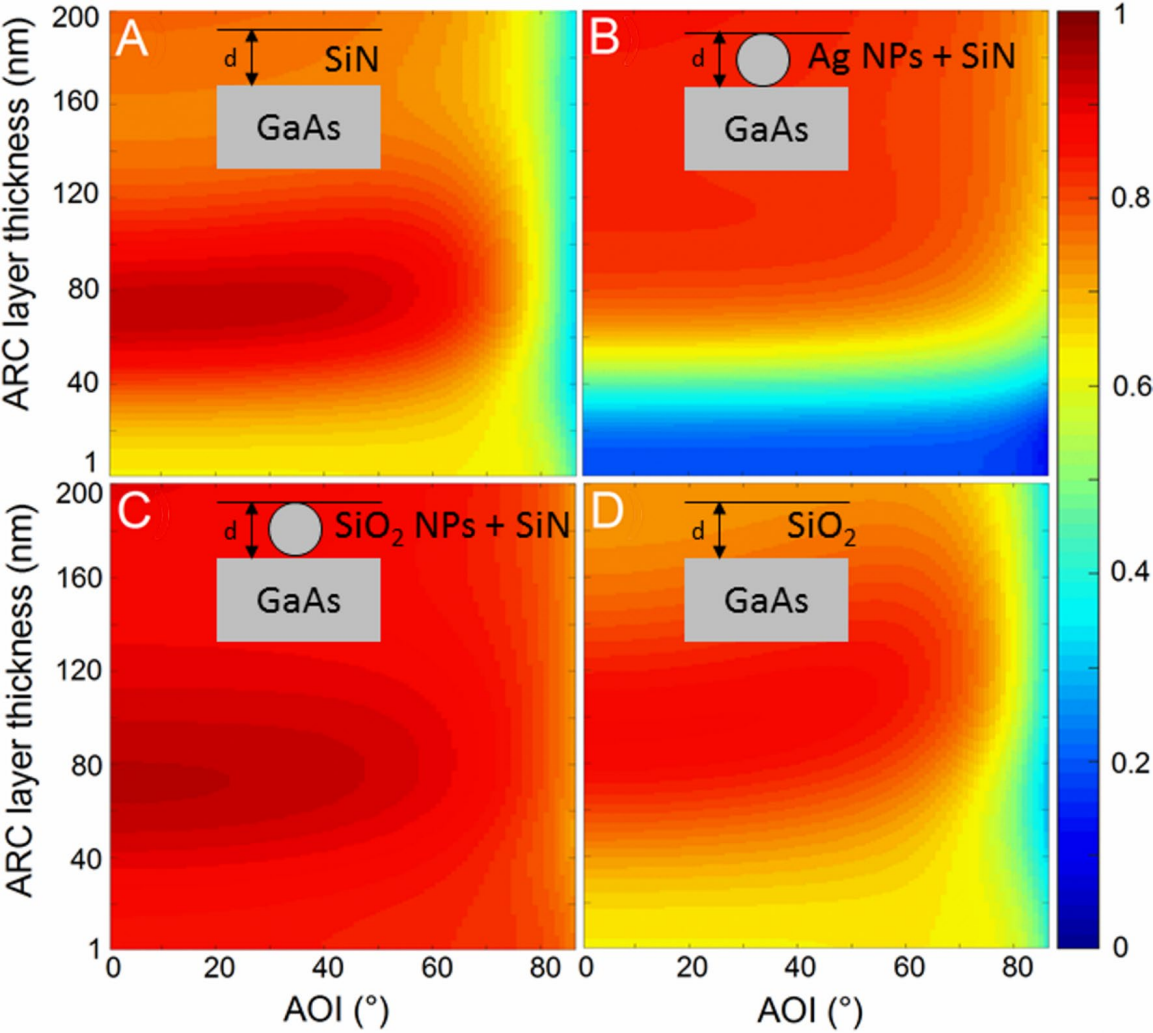
Weighted solar power transmittance ${(T}_{W})$ from (**A**) thin film SiN ARC, (**B**) hybrid ARC (Ag NPs + SiN), (**C**) hybrid ARC (SiO_2_ NPs + SiN), and (**D**) thin film SiO_2_ ARC. d: thickness of the ARC layer. The diameter of NPs is equal to the thickness of ARC layer. The contours of hybrid ARC (NPs + SiN) are for 70% surface filling density. 0° AOI corresponds to normal incidence.

The ARCs in solar cells are designed to minimize the reflectance and maximize the transmittance across the wavelength range of interest. Figure 2 shows the weighted solar power transmittance: $T_{w} = \mathop \smallint \limits_{300 nm}^{1200 nm} T\left( \lambda \right) S\left( \lambda \right) d\lambda /\mathop \smallint \limits_{300 nm}^{1200 nm} S\left( \lambda \right) d\lambda$, where $T\left( \lambda \right)$ and $S\left( \lambda \right)$ are the transmittance from the ARC layer and the intensity of AM1.5D solar spectrum at wavelength $\lambda$. The intensity of AM1.5D solar spectrum is taken from Ref.^25^. Thin film SiN ARC layer shows high transmittance for the thickness 60–80 nm over a wide range of AOI (Fig. 2A). When the ARC layer thickness increases, the transmittance decreases slowly at normal incidence, however with AOI, the change is drastic after 40° AOI. A fixed solar cell on a house roof, receives the sun light throughout a day at various AOI. Thus, the ARC should perform better at higher AOI. Embedding Ag NPs in the SiN ARC shows better performance at higher AOI (Fig. 2B). However, the performance is bad at normal incidence comparing to the thin film SiN ARC because metal NPs absorb the sunlight, and SiN layer doesn’t absorb^9^. Figure 2B also shows an expected behavior that metal NPs have large optical cross section than geometrical cross section^26^. Smaller Ag NPs (blue region in Fig. 2B) absorb almost all the sunlight incident upon it. Therefore, we replaced metal NPs (Ag NPs) with dielectric NPs (SiO_2_ NPs) because dielectric NPs do not absorb the sunlight in a narrow band spectrum as discussed in the beginning. The weighted solar power transmittance from hybrid ARC (SiO_2_ NPs + SiN) is shown in Fig. 2C. SiN ARC and hybrid ARC (SiO_2_ NPs + SiN) show nearly the same behavior for ARC thickness interval 60–80 nm at AOI 0°–40°. Furthermore, the improvement in transmittance from hybrid ARC (SiO_2_ NPs + SiN) is omnidirectional. For comparison, we also calculated the transmittance for 100% SiO_2_ thin film layer as ARC which shows a decrease in the transmittance at higher AOI (Fig. 2D). From the principle of single layer antireflection coating^13^, the refractive index of a perfect ARC should follow the following expression: $n_{ARC} = \sqrt {n_{Air} *n_{Substrate} }$. If we take $n_{Substrate} = 3.5$ (for GaAs or Si), $n_{ARC}$ equals to 1.87. The refractive index of SiN $(n_{SiN} )$ and SiO_2_ $\left( {n_{{SiO_{2} }} } \right)$ is 2.05 and 1.5 approx., respectively. Hence, $n_{SiN}$ is near to $n_{ARC}$, but $n_{{SiO_{2} }}$ is far from that. According to the effective medium theory^13^, embedding SiO_2_ NPs in the SiN dielectric matrix brings the effective refractive index nearer to $n_{ARC}$, therefore we obtained the best ARC performance in the hybrid ARC (SiO_2_ NPs + SiN).

**Figure 3:**
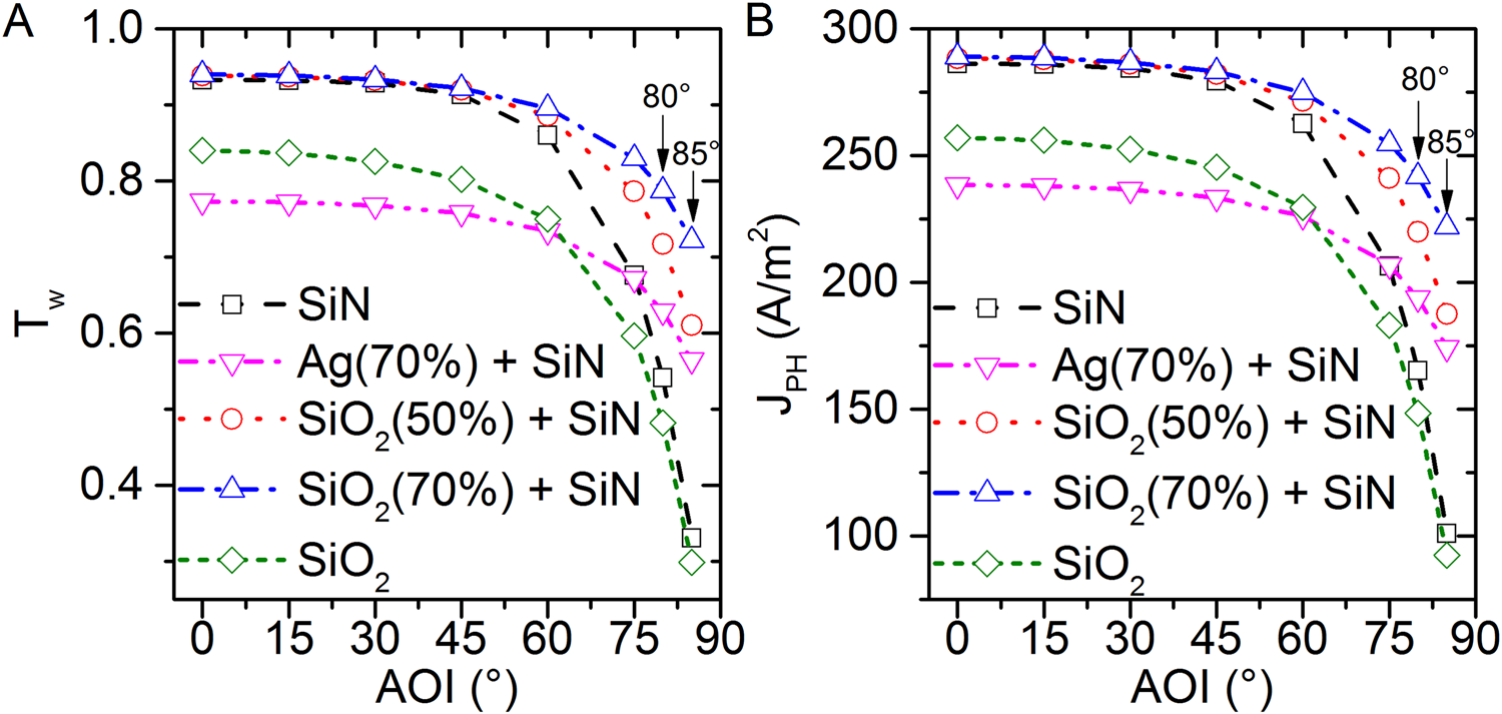
(**A**) Weighted solar power transmittance ${(T}_{W})$, and (**B**) photocurrent density ${(J}_{PH})$ for 70 nm thick ARC layer. The diameter of NPs are equals to the thickness of ARC layer. Ag (70%) + SiN : the surface filling density of Ag NPs is 70% in the hybrid ARC.

In the solar cell application, photocurrent density $(J_{PH} )$ is more commonly discussed property of a solar cell than the transmittance. So, we calculated $J_{PH} = q*\mathop \smallint \limits_{300 nm}^{870 nm} T\left( \lambda \right) PFD\left( \lambda \right)IQE\left( \lambda \right) d\lambda$, where $q$ is the electronic charge; $PFD\left( \lambda \right)$ and $IQE\left( \lambda \right)$ are the photon flux density and the internal quantum efficiency at wavelength $\lambda$. The photon flux density is taken from Ref. ^27^. Operating wavelength is taken from 300 to 870 nm because GaAs absorbs the sunlight in this wavelength range. We assumed that all the photons which reaches to the GaAs layer, got absorbed; hence $IQE\left( \lambda \right) = 1, for all \lambda$. We plotted the weighted solar power transmittance $(T_{W} )$ for an optimum condition of the hybrid ARC (SiO_2_ NPs + SiN) at the surface filling density $f = 0.70$ and $d = 70 nm$ (Fig. 3A). $J_{PH}$ curves follow the same trend as $T_{W}$ curves. $J_{PH}$ is almost equal for the SiN ARC structure and the hybrid ARC (SiO_2_ NPs + SiN) structure till 45° AOI, and after that the curves split (Fig. 3B). At 85° AOI, the improvement is as much as ca. 120% for the hybrid ARC (SiO_2_ NPs + SiN) structure comparing to the SiN ARC. We also plotted $J_{SC}$ and $T_{W}$ curves of the hybrid ARC (SiO_2_ NPs + SiN) at $f = 0.50$ (red curve in Fig. 3B), which is in between of the SiN ARC and the hybrid ARC (SiO_2_ NPs + SiN) at $f = 0.70$. This means when $f$ goes to zero, the hybrid ARC acts like the SiN ARC (as expected). With the help of Fig. 3B, we also calculated a net normalized photocurrent density over the course of a year w. r. t. the normal incidence by using the following expressions at different locations which is summarized in Table 1.

1 $$ J_{{PH\left( {in one day} \right)}} = \frac{1}{{day duration \left( {in minutes} \right)}}\mathop \smallint \limits_{sunrise}^{sunset} norm.J_{PH} dt\left( {in minutes} \right) $$

**Table 1:** Normalized photocurrent density over the course of a year (2020) at different locations (Lat.: latitude; Opt.: optimum tilt angle; Hybrid ARC: SiO_2_ (70%) + SiN).

ARC type	Munich, Germany (Lat. = 48.13°)		Chennai, India (Lat. = 13.07°)		Perth, Australia (Lat. = − 31.93°)		Antofogasta, Chile (Lat. = − 23.43°)	
	Opt. = 33°	45°	Opt. = 13°	45°	Opt. = 27°	45°	Opt. = 22°	45°
SiN ARC	0.85	0.85	0.87	0.82	0.87	0.85	0.87	0.84
Hybrid ARC	0.89	0.88	0.93	0.88	0.92	0.89	0.92	0.89
% Increase	4.71	3.53	6.89	7.32	5.75	4.71	5.75	5.95

To calculate over the course of a year, average $J_{PH}$ is calculated for each of the day of a year using Eq. 1 and summed over. The summation is divided by total no. of days in a year. AOI of the sunlight at the GaAs layer changes at every minute of the day and was calculated by the formula given in Ref.^28^. The GaAs layer is assumed to face due south in the Northern Hemisphere, or due north in the Southern Hemisphere. We compared the normalized photocurrent density at two different tilt angles cases for the given location: optimum tilt angle^29^ and 45°. The sun position calculation was done with a freely available MATALB code^30^. The improvement in hybrid ARC is clearly noticed at every location. The change due to hybrid ARC is more when the tilt angle is far from the optimum tilt angle (Table 1). Overall, the hybrid ARC has shown improvement in the antireflection property and recommended to use in solar cells.

To the best of our knowledge, the simulated device structure in this study has not been yet reported theoretically or experimentally. Other structures such as NPs layer on a solar cell absorber^1,8,10–12,14,19^, NPs layer on a thin film ARC layer with an absorber layer^13,16,19^ or NPs fully buried inside a thin film ARC layer with an absorber layer^31^ have been studied extensively. Ha et al. have reported more than 8% enhancement in the current density with 70% surface coverage by SiO_2_ NPs on a GaAs solar cell at normal incidence comparing to a bare GaAs solar cell^11^. The enhancement is also noticed at non-normal AOI. However, the sunlight still faces high reflection in the vicinity of NPs due to the host medium (air) at normal and non-normal AOI. In our device structure, the vicinity is filled with dielectric SiN host medium. At normal incidence, both the thin film SiN ARC and SiO_2_ NPs show approximately equal $T_{W}$ (a small insignificant increase at 0° AOI is noticeable in Fig. 3). But, due to constructive interference at the front surface, the thin film SiN ARC show decrease in $T_{W}$ at higher AOI whereas NPs don’t show this behavior, as reported in Ref. 23^23^. As a result, enhancement in $T_{W}$ at higher AOI has been noticed for hybrid ARC (SiO_2_ NPs + SiN) case (Figs. 2C, 3). Starowicz et al. have grown an ARC structure similar to ours in which metal NPs are successfully embedded in a TiOx thin film layer^32^. The ARC shows 5% increase in the short circuit current at normal incidence. In this case, the TiOx film is deposited with sol–gel method. Similarly, SiN thin film can also be deposited as reported in Ref.^33^, nevertheless no results of SiN deposition on a solar cell device structure have been reported by using this method due to complexity in synthesis and non-uniformity in the SiN structure. The controlled size homogeneity and distribution of NPs are also a difficult task, but it can be approximated on a device surface by density distribution calculation^31,34^. Lesina et al. modelled and characterize Ag NPs embedded in a SiO_2_ ARC^31^. In this case, Ag NPs are fully buried inside the thin ARC layer. Furthermore, NP’s size and surface coverage have been obtained with the density distribution calculation using ImageJ software. From the fabrication point of view, metal NPs can be synthesized by evaporation of its metal thin films, followed by thermal annealing^34–36^. However, NPs are also commercially available with various manufacturer such as nanoComposix, Inc.^10^. Hence, our simulated device structure can be studied experimentally in future and can also be used to guide future experimental design for wide angle antireflection coating and to predict its performance.

In summary, we performed an analytical study which suggests that a combination of $\lambda /4$ coating ARC with dielectric SiO_2_ NPs (hybrid ARC) improves the photocurrent density in a GaAs layer at higher AOI. We obtained more than 100% enhancement in the photocurrent density at 85° AOI. Sunrise to sunset calculation of the normalized photocurrent density have also shown improvement in the hybrid ARC case. In future, solar cells integrated into buildings, cars, etc. might become more important. In these cases, the panels are typically not oriented toward the sun and they have to deal with AOI far from the normal incidence. Conventional $\lambda /4$ coating ARC such as thin film SiN ARC is generally optimized for a certain AOI (typically close to normal incidence). Therefore, the hybrid ARC gives a route to alleviate the unwanted reflection at higher AOI and increase the overall efficiency of a solar cell.

## Methods

The simulation study is performed using an analytical model^23^ which is a combination of Transfer matrix method (TMM), Dipole model and Mie theory. A self-generated MATLAB code is used to solve Mie theory for the scattering efficiency of metal and dielectric NPs. Using the dipole model, an angular distribution of dipole radiation by a NP in the neighboring substrate is obtained which is later used to calculate the total diffuse scattering (reflected and transmitted) by a NP. The total transmittance is calculated with a linear summation of specular transmittance and diffuse transmittance in which the specular transmittance is calculated with the TMM method and the diffuse transmittance is calculated by the diffuse scattering of NPs.

Assuming a perfect absorber layer (IQE = 1) based solar cell device, the transmittance obtained from the analytical model is used to calculate the photogenerated current density. For the angle of incidence (AOI) of the sunlight on a solar cell surface, the sun’s position is calculated by a freely available MATLAB code^30^.
